# Cell Culture on MEMS Platforms: A Review

**DOI:** 10.3390/ijms10125411

**Published:** 2009-12-18

**Authors:** Ming Ni, Wen Hao Tong, Deepak Choudhury, Nur Aida Abdul Rahim, Ciprian Iliescu, Hanry Yu

**Affiliations:** 1 Institute of Bioengineering and Nanotechnology, 31 Biopolis Way, The Nanos, #04-01, Singapore 138669, Singapore; E-Mails: mni@ibn.a-star.edu.sg (M.N.); whtong@ibn.a-star.edu.sg (W.H.T.); deepakc@ibn.a-star.edu.sg (D.C.); hyu@ibn.a-star.edu.sg (H.Y.); 2 NUS Graduate School for Integrative Sciences and Engineering, Centre for Life Sciences (CeLS), #05-01, 28 Medical Drive, Singapore 117456, Singapore; 3 Department of Physiology, Yong Loo Lin School of Medicine, National University of Singapore, MD9 #03-03, 2 Medical Drive, Singapore 117597, Singapore; 4 NUS Tissue-Engineering Programme, DSO Labs, National University of Singapore, Singapore 117597, Singapore; 5 Department of Mechanical Engineering, Massachusetts Institute of Technology, Cambridge, MA 02139, USA

**Keywords:** cell culture, MEMS platforms, biocompatibility, biomaterials

## Abstract

Microfabricated systems provide an excellent platform for the culture of cells, and are an extremely useful tool for the investigation of cellular responses to various stimuli. Advantages offered over traditional methods include cost-effectiveness, controllability, low volume, high resolution, and sensitivity. Both biocompatible and bio-incompatible materials have been developed for use in these applications. Biocompatible materials such as PMMA or PLGA can be used directly for cell culture. However, for bio-incompatible materials such as silicon or PDMS, additional steps need to be taken to render these materials more suitable for cell adhesion and maintenance. This review describes multiple surface modification strategies to improve the biocompatibility of MEMS materials. Basic concepts of cell-biomaterial interactions, such as protein adsorption and cell adhesion are covered. Finally, the applications of these MEMS materials in Tissue Engineering are presented.

## 1. Introduction

The development of Micro-Electro-Mechanical Systems (MEMS) technology and its integration into complex systems for biological applications has generated a new field of study, called “BioMEMS” [1–4]. MEMS technology offers the advantage of building two dimensional (2D) or three dimensional (3D) structures, with micrometer-scale precision, incorporating different materials with differing chemical or physical properties such as polymers, metals, and dielectric materials. The complexity of solutions that can be generated by MEMS technology correlates with the requirements from biomedical applications, resulting in a large range of BioMEMS applications, from micro total analysis systems (μTAS) [5], implants for drug delivery [6], biosensors [7], stents [8], immunoisolation devices [9], microneedles [10] or injectable micromodules [11], up to pacemakers and cardiology devices [12].

Due to the increasing ease by which such powerful tools are available, life scientists and bioengineers have started to use MEMS as a platform for cell culture in order to better answer some fundamental biological questions [13]. For instance, researchers at the University of Washington are studying single cells using MEMS tools because conventional cell culture systems provide only an average from a population of cells [14]. Skelley *et al.* [15] recently demonstrated an efficient method for cell pairing and fusion in a microfluidic device, a very important aspect in somatic cells reprogramming research. This review covers MEMS applications in Tissue Engineering. The first part is dedicated to materials biocompatibility while in the second part, various applications are presented.

## 2. Protein Adsorption for Cell Attachment

Culture of adherent cells involves attachment of these cells onto a surface. This phenomenon involves an adhesive interaction between the cell and substrate. In order to facilitate this interaction, a layer of protein is usually adsorbed onto the surface of the substrate. Various measurements have been used to demonstrate the process of protein adsorption onto culture surfaces. Mahmood *et al.* [16] used X-ray photoelectron spectroscopy (XPS), a surface analysis tool, to show that the nitrogen signal, an indicator of the amine bonds of organic molecules, was significantly higher on bioactive glass after immersion in a cell culture medium than in a phosphate buffered solution (PBS). This demonstrates the adsorption of proteins from the culture medium onto the glass surface. Steele *et al.* [17] measured the amounts of vitronectin (Vn) and fibronectin (Fn) which adsorbed from the fetal bovine serum (FBS) component of the culture medium onto Primaria^™^ (the material used for cell culture flasks) and tissue culture polystyrene (TCPS, the material used for cell culture plates). It was found that Primaria adsorbed two- to three-fold more Fn than TCPS, but adsorbed similar amounts of Vn from medium containing FBS. The difference of protein adsorption onto different materials subsequently affects the number of cells adhered to these materials, and the strength of adhesion.

As protein adsorption is a very important factor when studying the interaction between the cells and biomaterials, various methods have been developed to quantify the amount of adsorbed protein, including radio-labeling [18–20] and fluorescence-labeling [21,22] of proteins. Other surface analysis techniques used for this purpose include surface plasmon resonance (SPR) [19,22–24], secondary-ion mass spectroscopy (SIMS) [19,25–27], and XPS [27].

Cellular behavior is affected not only by the amount of adsorbed protein, but also the orientation and conformation of the protein. For example, Fn, a 440-kD glycoprotein, is well known to be involved in cell adhesion [28]. The argininie-glycine-asparagine (RGD) sequence is essential for Fn binding to the transmembrane integrin receptor. Iuliano *et al.* [21] showed that surface hydrophobicity of a biomaterial has an effect on the conformation of this cell binding domain of Fn and consequently, Fn conformation change influenced bovine aortic endothelial cell (BAEC) adhesion. Antia *et al.* [29] have used fluorescence resonance energy transfer (FRET) to reveal the conformational changes of Fn molecules. Alternatively, Cheng *et al.* [30] used another tool, Fourier Transform Infrared Spectroscopy Attenuated Total Reflectance (FTIR/ATR), to study the conformational change of Fn on self-assembled monolayers.

## 3. Cell Adhesion

Adhesion of cells onto the culture surface precedes cell spreading, cell migration, and cell differentiation. Methods of quantifying the number of attached cells include direct microscope visualization and cell counting, colorimetric assays such as using toluidine blue dye [31], measuring the concentration of an intracellular enzyme (e.g., lactate dehydrogenase (LDH) assay [32]), and using PicoGreen assay, a DNA based analysis method [33]. Besides measuring the number of attached cells, it is sometimes necessary to find out the attachment strength of the adhered cells. Typically, centrifugation or fluid flow is used to measure the force. Garcia *et al.* [34] used a spinning disc device to measure cell detachment while Qin *et al.* [35] used a micropipette technique to measure the force of cell-surface adhesion. Further parameters that influence cell adhesion on MEMS materials are described below.

## 4. Biocompatibility of MEMS Materials

The chemical structure and surface property of the MEMS materials determine their biocompatibility through protein adsorption and cell adhesion. Meanwhile, the surface chemistry (functional group, surface charge, hydrophilicity/hydrophobicity), surface roughness, and surface topography may first affect protein adsorption, and sequentially affect the cell’s adhesion onto the materials.

### 4.1. Surface Chemistry

#### 4.1.1. Surface Functional Groups

Over a decade ago, Mrksich and Whitesides [36] wrote a review on the use of self-assembled monolayers (SAMs) as a model to understand the interactions of man-made surfaces with proteins and cells. SAMs provide model surfaces with different surface functional groups, such as hydroxyl (OH), carboxyl (COOH), amine (NH_2_), and methyl (CH_3_) groups. In particular, Keselowsky *et al.* [37] have used such model surfaces to investigate the effects of surface chemistry on Fn adsorption, integrin binding, and cell adhesion. Cell adhesion strength to Fn-coated SAMs was found to be in the following order:

$$\text{OH}>\text{COOH}={\text{NH}}_{2}>{\text{CH}}_{3}$$

However, so far there is no conclusion on which specific functional group is able to render biomaterials more biocompatible.

#### 4.1.2. Surface Charge

The effect of surface charge on IgG antibody orientation was investigated using NH_2_ (positively charged) and COOH (negatively charged) terminated SAMs as model surfaces [38]. The authors showed that better antibody orientation was achieved on the positively charged surface. Additionally, surface charge can be determined by Zeta-potential measurements.

#### 4.1.3. Surface Hydrophilicity and Hydrophobicity

Allen *et al.* [14] manufactured a homologous series of copolymer films that subtly vary in terms of surface hydrophobicity. Using cell-lines such as HeLa (epithelial) and MRC-5 (fibroblast), they found that cell number increased with increasing hydrophilicity. Surface hydrophilicity and hydrophobicity can be determined by contact angle measurements.

### 4.2. Surface Roughness

Although most of the cell adhesion enhancements conducted in MEMS is done by chemical surface modification, the role of microtopography in controlling cell adhesion is gaining importance. Early work in this area was conducted by randomly roughening fabricated surfaces using sandblasting, grinding, and electropolishing. It has been shown that when the surface is randomly modified, submicron-scale roughness significantly affects cells adhesion [39–42].

These observations were further refined by investigation of the effects of uniform submicron roughness modifications, giving further control over surface geometry. In one study, HepG2 (human hepatocellular carcinoma cell line) exhibited a 200% increase in adhesion after poly(glycolic-*co*-lactic)acid (PGLA) copolymer substrates were modified to give a uniform distribution of 3.1 ± 1.5 μm micropores [39]. In another study, osteoblasts cultured on 9 to 29 nm nano-pits or nano-islands also show enhanced attachment compared to a smooth surface [43].

### 4.3. Surface Topography

Apart from creating rough surfaces, cell adhesion can also be optimized by fabricating unique micro- or nano-patterns targeted to specific cells, or by increasing the surface area on which cells can attach. In the work of Deutsch *et al.* [44], neonatal rat primary cardiac myocytes were cultured on 5 μm tall micropegs, which allowed a more perpendicular attachment to the membrane. As a result, the number of myocytes adhered to pegged membranes was increased 4-fold in comparison with untextured membranes.

Nevertheless, more systematic studies are still required to convert such knowledge into useful applications. Specifically, there is no one common adhesion trend across various cells and cell lines. For example, fibroblasts have been reported to show reduced adhesion in nano-pit structures [45] while osteoblast-like cells, OCT-1, show an increase in attachment on pit-patterned surfaces [46]. Furthermore, the scale of the microtopography needs to be optimized for different types of cell adhesion. For instance, human fetal osteoblastic cells show enhanced cell adhesion in lower nano-island surfaces (11 nm in height) than higher nano-island surfaces (38 nm and 85 nm) [47]. Fibroblast adhesion is higher on 13 nm islands but lower on 95 nm islands when compared to smooth surfaces [48–50].

As another example, Zinger *et al.* [51] prepared titanium disks with cavity diameters of 10, 30 and 100 μm. They showed that when cultured with MG63 cells (osteoblasts), the cell number, osteoblastic differentiation (alkaline phosphatase; osteocalcin) and local factor levels (TGF-β1; PGE-2) varied with microarchitecture.

## 5. Enhancement of Biocompatibility through Surface Modification

Several surface modification approaches have been applied to enhance the biocompatibility of MEMS materials, including plasma deposition, ultra-violet irradiation, gamma irradiation, ion-beam irradiation, chemical vapor deposition (CVD), covalent modification, and protein immobilization.

### 5.1. Plasma Deposition

Plasma is a mixture of electrons, neutral radicals and ions with high energy, in which positive and negative charges are present in equal amounts. Sources of plasma include glow discharges, radio frequencies, and gas arcs. A brief review of plasma deposition for biomedical applications can be found in [52]. Poly(*N*-isopropylacrylamide) (PNIPAAm), has been widely utilized as surface coating for biomaterial and Tissue Engineering applications [53–55]. PNIPAAm exhibits temperature responsive surface properties, *i.e.,* the surface is hydrophobic in the cell culture condition at 37 °C and changes to hydrophilic below the lower critical solution temperature (LCST) at about 32 °C. Cell adhesion and cell detachment on PNIPAAm can be controlled by switching the temperature. One way to create a PNIPAAm coating is plasma deposition [56]. Cheng *et al.* used this approach to pattern cells on a photolithographically fabricated microheater array. The plasma deposition approach has been applied to polymerization of other polymers, such as hexamethyldisilazane (HDMS) [57], ethyl ether, methyl or ethyl acetate, acetaldehyde, acetone and 2-propanol [58]. A new instrument was developed that combined electrospray ionization with a radio frequency (RF) plasma [59]. This instrument was used successfully to deposit a number of high molecular weight active biomolecules including the polysaccharide, sodium hyaluronan (HA).

### 5.2. Irradiation for Grafting Polymerization

Irradiation can generate free radicals, which act as sites for graft polymerization. Ultraviolet and gamma irradiation are two commonly used methods. To enhance cell adhesion, Ebara *et al.* [60] reported surface coating PNIPAAm on poly(dimethylsiloxane) (PDMS) by UV mediated graft polymerization. Other polymers, such as acrylic acid (AA), acrylamide (AM), dimethylacrylamide (DMA), 2-hydroxyethylacrylate (HEA), and poly(ethylene glycol)monomethoxyl acrylate (PEG) were also grafted on PDMS using UV radiation [61]. Photopolymerizable biomaterials provide us another type of useful template material for MEMS. Recently, Kloxin *et al*. [62] reported a novel photodegradable poly(ethylene glycol)-based hydrogel. This material is cytocompatible and can gel rapidly. Living cells can be encapsulated into the hydrogel. When shone with light, microchannels made by this hydrogel can be degraded, allowing the migration of encapsulated cells.

### 5.3. Covalent Modification for Protein Immobilization

Covalent bonding has been used to enhance cell adhesion. Leong *et al.* [63] sputtered a thin layer of gold on PDMS, functionally engineered the gold surface with a SAM, and bound collagen covalently to the SAM using Schiff based chemistry. After surface modification, cells were found to be attracted and adherent to the chemically modified PDMS. Immobilization of cell-adhesive proteins or oligopeptides, such as RGD, on the surface can also enhance cell adhesion. In another study, osteopontin was immobilized onto poly(2-hydroxyethyl methacrylate) (pHEMA) using a CDI (1,1-carbonyldiimidazole) chemistry [64]. Osteopontin is an extracellular matrix molecule involved in wound-healing processes. It also contains an RGD moiety. Cell adhesion studies showed that the number of BAECs attaching on pHEMA increased after the immobilization of osteopontin. Chen *et al.* [65] created patterned substrates with different shapes using micro-contact printing. Different extracellular matrix (ECM) proteins were coated onto the substrates. They found that cell shape governs the growth or death of individual cells regardless of the type of matrix protein or antibody to integrin used to mediate adhesion. Flaim *et al.* [66] developed an ECM microarray platform. Five ECM molecules (collagen I, collagen III, collagen IV, laminin, fibronectin) were mixed to achieve 32 combinations. The effects of these combinations on cell differentiation were studied. With this type of mircoarray platform, the synergic effects of ECM molecules on cell differentiation can be studied.

### 5.4. Chemical Vapor Deposition

Chemical vapor deposition (CVD) is a process which transforms gaseous molecules into a solid material which can form a thin film on the surface of a substrate. It is well-known that silicon nitride (Si_3_N_4_) deposited through plasma enhanced chemical vapor deposition (PECVD) or low pressure chemical vapor deposition (LPCVD) is a good material for cell attachment. The PECVD method has the advantage of a low deposition temperature and relatively fast process while the LPCVD technique is a high throughput technology. Si_3_N_4_ is the main material deposited using these processes, due to its suitability for cell culture [67]. The main reason for enhanced cell attachment is the presentation of NH_2_ groups on the thin film surface. Behind this, the gas precursors for the deposition of Si_3_N_4_ on PECVD and LPCVD are SiH_4_/NH_3_/N_2_ and DCS/NH_3_/N_2_ which explains the increased quantity of hydrogen presented in the film. Work by Neumann *et al*. [68] has shown however, that there still exist cytotoxic effects of Si_3_N_4_ ceramic samples (L929-cell culture model).

## 6. MEMS Materials

The many materials currently being used for MEMS devices generally fall into three categories, silicon and silicon-based materials, polymers, and metals.

### 6.1. Silicon and Silicon-Based Materials

Silicon is the basic material for microfabrication. For this reason its biocompatibility and the biocompatibility of related materials is of great interest to researchers. A complex study was carried out to investigate the biocompatibility of MEMS materials such as single crystal silicon, polycrystalline silicon, silicon dioxide, silicon nitride, single-crystal silicon carbide, titanium, and the photo epoxy SU-8 substrates [69]. The study shows that the above mentioned materials are not cytotoxic when tested *in vitro* using mouse fibroblasts. These materials are classified as non-irritants based on 1- and 12-week rabbit muscle implantations. The study also revealed that Si_3_N_4_ and SU-8 leached detectable nonvolatile residues in aqueous physiochemical tests. It can be concluded that there are few concerns regarding the use of these materials for *in vivo* or *in vitro* testing.

Amorphous silicon-membrane is considered a new type of membrane for use in hemodialysis [70]. Silicon chips bearing 1 × 1 mm arrays of approximately 10^4^ slit pores were fabricated via sacrificial layer techniques [71–73]. The pore structure is defined by deposition and patterning of a polysilicon film onto the silicon wafer. The critical submicron pore dimension is defined by the thickness of a sacrificial SiO_2_ layer, which can be grown with unprecedented control to within ±1 nm. The oxide layer is etched away in the final processing step to create the porous polysilicon membrane. Besides being used for ultrafiltration, this silicon-based membrane can also be used as a scaffold for renal proximal tubule cells. Renal tubule cells were observed to attach to single-crystal silicon and polysilicon chips when pretreated with ECM proteins. These cells retained surface markers characteristic of renal proximal tubule cells, including tight junction proteins like ZO-1. Trans-epithelial resistance (TER), a metric of tight junction formation necessary for proximal tubule function, was similar between monolayers grown on tissue culture plastic [74].

Porous silicon, fabricated from single crystal silicon by an anodization process, is a very interesting biomaterial. Its use in drug delivery is reviewed in [75] and [76] while the first report regarding its biocompatibility was performed in 1995 [77]. There are a significant number of studies regarding cell adhesion and culture [78–81], and protein adsorption [82–84] on porous silicon. A very interesting aspect is the biodegradability of porous silicon [85]. High-porosity mesoporous films made of porous silicon exhibited substantial dissolution *in vitro*, while the single crystal material is inert [86,87]. On the other hand, porous silicon made, low-porosity microporous films can induce hydroxyapatite growth *in vitro.* Hydroxyapatite is well known as the bone bonding material. *In vivo* studies regarding the tissue compatibility of porous silicon have also been attempted [88,89].

Silicon nitride [67,90–92] and silicon carbide deposited using CVD techniques are other materials that can be used for cell culture. Cytotoxic evaluation of the Si_3_N_4_ ceramics are evaluated in [93]. Silicon carbide deposited using PECVD can be used as an alternative of Si_3_N_4_ layers [94]. Cell adhesion on silicon carbide surfaces can be sensitively improved by dipping the samples in NH_4_F. It is also important to note that classical cleaning process of the silicon nitride/carbide layer in piranha (H_2_SO_4_/H_2_O_2_), or the exposure of the surface to different etching solutions used for definition of the MEMS structure, can have a strong influence on cell adhesion due to the chemical modification of the surface.

### 6.2. Polymers

Polymers used as MEMS materials include poly(methylmethacrylate) (PMMA), polyvinylchloride (PVC), polycarbonate (PC), polystyrene, polyurethane, and poly(dimethylsiloxane) (PDMS), though the bulk of use comes from PDMS devices.

#### 6.2.1. PDMS

One of the most widely used polymeric materials for MEMS is PDMS. Soft lithography, novelty adapted by the Whitesides group [95–97], is the most commonly used technique for the fabrication of PDMS chips. PDMS presents some important advantages such as rapid prototyping, cost-effectiveness, ease of visualization (transparent), good gas-permeability, excellent adhesion to glass and many substrates, and high fidelity of feature production when cast on microfabricated masters [52]. However, PDMS is very hydrophobic, which makes the micro-fluidic channel difficult to fill with aqueous solutions. Due to its high hydrophobicity, PDMS absorbs some organic solvents and some hydrophobic analytes, causing fouling of the material. Also, the aspect ratio of the features that can be generated in PDMS (depth of the trench/width of the trench) is 2:1, which is significantly lower than that of silicon (usually 20:1).

Some strategies have been applied to enhance the biocompatibility of PDMS [97,98]. For example, Mirzadeh *et al.* prepared PDMS samples with different crosslink density in order to track differences in molecular mobilities. Their results suggest that molecular mobility causes changes in cell behavior, with the optimum cell attachment and proliferation being dependent on the number and surface area of cells. In a review paper, Makamba *et al.* summarized seven major approaches for surface modification of PDMS:

1. Modification by exposure to energy.
2. Dynamic modification using charged surfactants.
3. Modification using polyelectrolyte multilayers.
4. Covalent modification.
5. Chemical vapor deposition.
6. Phospholipid bilayer modification.
7. Protein modification.

Various types of bioreactors composed of microstructured PDMS have recently been fabricated for perfusion culture of mammalian cells. Leclerc *et al.* [99] demonstrated the cultivation of fetal human hepatocytes (FHHs) in such a PDMS bioreactor. During a one-week perfusion culture in the PDMS bioreactors, cells showed good attachment and spreading, and reached confluence across the channels. Perfusion culture demonstrated better performance in comparison with static culture, in terms of albumin production, an important function of hepatocytes. This was significantly enhanced during FHH perfusion culture within the PDMS bioreactors by up to about four times compared with dish-level static culture. Similar results were seen when using the HepG2 cell line. Under perfusion conditions, HepG2 cell activity was doubled compared to static conditions.

Another type of PDMS bioreactor was used for cultivation of liver cells [100]. A PDMS membrane less than 20 μm thick, with 5 × 5 μm pores served as a scaffold for the attachment of cells. During the fifteen days of perfusion in these bioreactors, good cell attachment and subsequently cell reorganization was observed. Moreover compared to static cultures in tissue-culture-treated dishes, perfusion culture of primary rat adult hepatocyte showed enhanced albumin secretion and ammonium removal.

These new microbioreactors, which attempt to more closely mimic the *in vivo* liver architecture, appear to be very promising tools towards future applications in drug screening or liver Tissue Engineering.

Besides liver Tissue Engineering, PDMS-based microfluidic channels have been applied to other systems such as bone [101], blood vessel [102], nerve [103], and kidney [104] Tissue Engineering.

Mouse calvarial osteoblastic cells, MC3T3-E1, were seeded at 2 × 10^6^ cells/ml and cultured in microdevices under flow rates of 0, 5, and 35 ml/min [101]. These PDMS microdevices were fabricated with a 3D microstructured channel network for bone Tissue Engineering. Cells attached and proliferated well within the designed microdevices. Cell viability was around 85% up to 1 to 2 weeks for shear stress values under 5 mPa. The alkaline phosphatase (ALP) activity was enhanced under dynamic flow as compared to static cultures in PDMS coated Petri dishes.

Micro- to nanogrooved PDMS channels were used as substrates on which to culture BAECs in order to mimic the uniformly elongated endothelium in natural linear vessels [102]. The channel depth ranged from 200 nm, 500 nm, 1 μm, to 5 μm. Smooth surfaces served as control. They found that cell alignment was strongest for substrates with 1 μm deep channels at all culture times, namely 1, 4, 24, and 48 h. PDMS substrates engraved with micro- and nanochannels serve as a useful tool for investigating the topography-cell/cytoskeletal alignment interplay.

Li *et al*. [103] developed a PDMS microchip for monitoring the amount of catecholamines released from rat pheochromocytoma (PC 12) cells. By varying the concentration of PC 12 cells immobilized in microchannels, the authors were able to achieve a catecholamine release ranging from 20 to 160 mM. Catecholamine has a stimulus effect on neurotransmitter release.

#### 6.2.2. PMMA and Other Polymers

PMMA is another type of polymer used for MEMS applications. This material is transparent and commonly known as Plexiglass. PMMA films can be directly patterned using electron-beam lithography [104,105] or laser ablation [106]. PMMA can be surface modified by aminolysis reaction to introduce amine groups on PMMA for DNA purification [107]. PC and PS are also transparent, and their thin films can also be patterned with laser ablation [108]. PMMA, PC, and PS are all biocompatible materials frequently used as substrates for mammalian cell culture.

### 6.3. Metals

BioMEMS devices require electrodes which, in the case of implantable devices, can be in contact with the tissue. As a result the biocompatibility of these materials is an important aspect. The main materials typically used as electrodes in implantable BioMEMS devices are gold, platinum, or titanium. Gold and platinum have been used for a long time in dentistry, while titanium is known for its successful application in orthopedic implants. The biocompatibility of titanium is addressed in [109] and [69], which also discusses biocompatibility of other MEMS materials. Gold and activated carbon electrodes used for electrochemical sensors are biocompatible. Evidence showed that these electrodes maintain their properties even after four years of implantation in animals [110].

## 7. On-Chip Cell Culture

The culture of cells, whether at the macro-scale as in wells and plates, or at the micro-scale as in MEMS platforms, necessitate considerations of the dynamic nature of culture conditions. These include effects of diffusion and delivery of soluble biochemical molecules, waste removal, nutrient depletion, mechanical forces, and extracellular matrix remodeling. The beauty of MEMS platforms is the ability to control these parameters through engineering of specialized devices. Kim *et al*. [111] have written a comprehensive practical guide on the use of microfluidic perfusion culture as one example of addressing these issues. These and other systems that demonstrate successful cell-handling solutions are presented below.

### 7.1. Microfluidics-Based Chips

**Figure 1.**
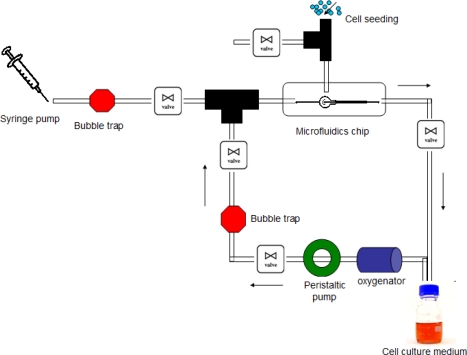
Schematic diagram of a perfusion system.

It is known that cells respond to spatial and temporal cues in their microenvironment. MEMS technology can be used to develop microfluidics interfaces that can mimic physiological conditions [112–114]. Microfluidic channel-based systems for cell culture have been developed where the cells have been successfully shown to grow on glass and PDMS, both rigid 2D substrates [112,115–117]. Researchers have also been able to successfully culture and elucidate the development of mammalian embryos in microfluidics chips [118–121]. However, 2D culture does not accurately represent *in vivo* conditions as it is lacking in terms of cell-ECM interaction, cell-cell interaction, soluble factors and mechanical forces in 3D [122,123]. Therefore, various methods have been developed to generate 3D environments including the use of scaffolds [124–126], bioreactors [127], and microstructured channels [101]. Toh *et al.* [128] have developed a 3D microfluidic channel-based cell culture system (3D-mFCCS) that facilitates the formation of cell-cell and cell-matrix interactions on the chip. A perfusion based culture can help in controlling the microenvironment of cells, through controlled delivery of biochemical factors, removal of waste and shear stress application through fluid flow [111]. An example of such a perfusion system used in our group is shown in Figure 1. In an effort towards realizing the ambition of human-on-chip, Zhang *et al*. [129] cultured multiple cell types in a fludically linked fashion. Compartmentalized microenvironments were achieved on the chip by control release of different growth factors, for example TGF-β1, by means of gelatin microspheres. Apart from mammalian cell lines and primary cells [130], microbes have also been grown in microfabricated platforms [130–133].

### 7.2. Cell Stimulation on Microfluidic Chips

Cells can be exposed to shear stress, pressure, and stretch and have been shown to be stimulated if experiencing fluid flow comparable to physiological blood flow [101,127,134]. Abhyankar *et al.* [135] proposed a system that can create stable chemical gradients without fluid flow. They used this device for cell-signaling applications, such as neutrophil chemotaxis. The Kamm group has also developed a microfluidic system that sustains concentration gradients and allows single- and multi-cell culture under shear stress [136–138]. As another example, an osmosis-driven pump was used to obtain a stable and wide concentration gradient profile [139]. They studied the behavior of human mesenchymal stem cells (hMSCs) by using this chemical gradient system. Other examples of temperature [140] and chemical gradients have also been used to study the embryonic development in *Drosophila* [141], the effect of colchicine on myoblasts [142], organelle movement in cells [143], and cell differentiation [144]. Cell stimulation has been achieved by various modalities including dielectrophoretic forces [145,146], bi-axial stretching [147], deflection [148], and cyclic mechanical stimulation [149].

### 7.3. Cell Characterization and Single Cell Analysis

The predominant single cell analysis method to date utilizes fluorescent probes and image-based techniques. However single cell analysis with MEMS technology [150,151] has enabled researchers to probe their system in a variety of other ways. For single-cell analysis of yeast, mammalian cells, and fungal pores in a microfluidic system, Palková *et al.* developed a pressure-driven chip-based method [152].

BioMEMS devices have been used extract, recover and analyze mRNA [153,154], amino acids [153], and proteins [155] from the cell lysis. In the work of Dittami *et al.*, sensory hair cells isolated from the cochlea of the mammalian inner ear were analyzed using electric impedance spectroscopy and electrochemical analysis [156]. Poenar *et al.* developed a microfluidic system for the analysis of single cells [157]. They integrated the steps of cell sampling, single cell loading, docking, lysing, and capillary electrophoretic (CE) separation with laser induced fluorescence (LIF) detection. Di Carlo *et al.* have designed a microfluidic-based dynamic single cell culture array [158]. This system allows arrayed culture of individual adherent cells with dynamic control of the fluid perfusion, creating uniform environments for individual cells. Cell membrane permeability can been enhanced by electroporation on chip [159–161], facilitating the manipulation of genetic, metabolic, and synthetic contents of single targeted cells at specific loci on a chip-based device. Pathogen and disease detection is also possible by doing PCR amplification on chips [161–163].

### 7.4. Cell Trapping and Sorting

Various types of cell separation methods have been developed, based on mechanical forces, dielectrophoresis (DEP), optical interactions, magnetic interactions and biochemical interactions [164]. Different cell types from blood have been shown to be trapped by microfabricated mechanical filters [165,166]. Three types of electric fields have been used to stimulate biological entities on chips: (a) a DC field for electrophoresis of charged particles, (b) dielectrophoresis, which is a non-uniform ac field, and (c) a combination of both of the above [167]. Bacterial, yeast and mammalian cells have been successfully separated using the DEP [168,169]. For optical methods, fluorescent sorting in microfluidic devices have been demonstrated, and a microfabricated fluorescence-activated cell sorter (mFACS) has been developed [170], which provides higher sensitivity and no cross-contamination at a lower cost. Other microfluidic cell sorting technologies developed include devices for high content cell analysis and sorting, impedance spectroscopy for cell sorting, magnetic cell sorting (MACS) [171], and selective cell lysis to biochemically separate the blood cell types [172,173].

### 7.5. Biosensors (Diagnostics)

A biosensor is a device used for the detection of specific cellular biochemical outputs mediated by isolated enzymes, organelles, whole cells, tissues, or immunosystems. Detection is usually achieved through electrical, thermal or optical signals [174], though detection through optical (colorimetric, fluorescent or luminescent) and electric (changes in impedance or electric potential) signals are more widely used. The use of MEMS technology in building biosensors has made point of care diagnostics easier. A whole array of biosensors has been developed for measuring various compounds in the body like blood gas [175,176], glucose [177,178], ethanol [179], cholesterol [180], uric acid [181,182], lactate [183,184], and pH [184,185]. Biosensors have also been developed to analyze the various components of blood including whole blood analysis [186], haemoglobin [187,188], differential blood cell counters [189], and blood ketone analysis [190]. Microbial biosensors detect disease-causing pathogens [191,192]. Many immunosensors have been fabricated to detect specific kinds of proteins by antigen / antibody binding events [193,194]. The idea of DNA sensors has also been realized [139–143,153]. Huang *et al.* [195] made use of an impedimetric immunosensor for high-throughput screening of liver fibrosis markers, lamin. In a more recent work, a biosensor and drug delivery module were integrated onto a microfabricated system [196]. The same group has also reported the design of a CMOS BioMEMS which demonstrates the possibility of integrating multiple type biosensors [197].

## 8. Precise Surface Patterning Using Microtechnology

The ability of MEMS to precisely modify and pattern a surface up to micrometer or even nanometer scale has opened up a whole new dimension for cell culture. Research has been conducted to study the effects of topography and microtexture on the dynamics of cell behavior in culture. Considering that ECM *in vivo* is made up of nanoscale structures [198], micro- and nanotopography created by MEMS is potentially able to recreate a life-like geometry that might extend biomimetic cues to the cell culture system.

### 8.1. Controlling Cell Orientation

Most cell culture studies on microtextured surface inevitably include cell orientation discussions, and hint at its profound effect. The effect is especially notable with grooved surface. Numerous studies have shown that most cell types such as myocytes [44], fibroblasts [199], and osteoblasts aligned their shape and elongated in the direction of the grooves.

Similar to cell adhesion, the scale of the grooves also plays an important role in determining cell alignment. It has been found that the cell orientation generally increases with increasing grove depth and decreases with increasing grooves width [200,201]. It has also been proposed that it is the aspect ratio of depth and width of gratings that affects the contact guidance behavior [202]. However, the range of topographic scale that induces contact guidance is still a debatable matter. One study reported that human dermal fibroblasts and human umbilical artery smooth muscle align poorly in grooves less than 1 μm in depth [203]. On the other hand, bovine corneal epithelial cells align parallel to substratum nanogrooves as shallow as 14 nm [204].

The directional growth of cells as determined by the topography has its own significant impact on Tissue Engineering. Firstly, organized cells allow one to recreate tissue architecture in a reproducible manner [205]. More importantly, a topography closely mimicking *in vivo* patterns might direct the cells to be organized into life-like tissue. When culturing osteoblast-like cells on nanogrooves, it was found that not only the cells and actin stress fibers were aligned and elongated along the direction of the nanogrooves, but the alignment of collagen matrix was also influenced by underlying nanogrooves. These results suggested that the alignment of bone tissue formed depends to some extent on nanoscale cues in the longitudinal direction [206].

### 8.2. Controlling Cell Behavior

Apart from cell adhesion and cell orientation, other aspects of topography-influenced cell behavior is less studied. Despite the lack of general trends and systematic studies on this issue, work that focus on proliferation, differentiation, and cell superstructure are reviewed here to suggest some of the potential effects that can be induced by surface patterning.

In terms of proliferation, most of the studies found that micro or nanostructure is often associated with a decrease in proliferation. Studies have been performed with fibroblasts [207], endothelial cells [208], osteoblasts [209], and even stem cells [210]. However, there are also contradictory results which show that topography has no influence over proliferation [46], and even increases proliferation of certain cell types [211]. As of now, it seems that there are still no obvious trends to predict the effect of surface geometry on proliferation and the interplay of geometry and the target cells. This could mean that effects are system- and cell-specific.

The general decrease in proliferation in stems cells cultured on patterned surfaces prompted some researchers to investigate whether it will induce differentiation in stem cells. Although the research in this field is still in its early stage, there are some promising results that support such a hypothesis. Yim *et al.* [212] showed that hMSCs can be differentiated into neuronal-like cells when cultured on a nanograting. Another study demonstrated the differentiation of osteoblasts from mesenchymal stem cells [213].

It has been shown that certain cell-surface pattern interaction is able to increase cell-cell interaction and subsequently alter the cell culture superstructure. Fabricated micropegs increase the surface area for myofibrils stacking [44]. As a result, myocytes cultured on micropegs are significantly higher than cells grown on a flat membrane, thus mimicking the cylindrical shapes found *in vivo*. On the other hand, human endothelial progenitor cells cultured on nanograting were organized into a multicellular band structure, instead of the confluent monolayer on the flat surface [214].

The precise mechanism of how microtopography induces the above-mentioned cell behavior and their direct relationships are still unclear. However, work has been conducted to analyze the effect of surface patterning on gene expression profiles to give an insight into the underlying molecular mechanism [215,216]. It is generally speculated that the initial effect of surface patterning affects individual cell cytoskeleton organization and focal adhesion formation [217]. Other effects taking place, for example percolation, suggests that cytoskeletal restructuring would result in changes of cytoskeletal-linked G-protein and kinase signaling and subsequently affect downstream biochemical pathways.

## 9. Co-Cultivation Using MEMS Platforms

Heterotypic cell interactions are important for optimal cell growth, migration, and differentiation. The demonstrated physiological importance of interactions between parenchymal cells and non-parenchymal neighbors has fueled attempts to replace and recover tissue functions through Tissue Engineering [218,219]. Effort towards this end has resulted in many works on co-culturing different type of cells together. These used to be performed by seeding cells separated by filters or varying the seeding density ratio. In the first instance, direct cell interaction is not possible, prohibiting cell-cell junctional interaction. In the second instance, there is no control over the spatial distribution of the different cell types. In contrast, the development of micro and even nanoscale MEMS devices has allowed up to micrometer resolution scale of cell culture patterning [220,221].

### 9.1. Co-Cultivation by Chemical Patterning

The most common technique employed in MEMS for cell patterning is to fabricate a layer of chemical patterns on a substrate. This is done in two steps. In the first step, chemicals or biomolecules are patterned using micro-contact printing or photolithography. This layer of chemicals will mediate the adhesion of the first cell type. The second cell type can be attached to the remaining surface by serum-mediated non-specific adhesion. Such a technique was used to co-culture hepatocytes and 3T3 fibroblast by Bhatia *et al*. [222].

A similar but more complex variation of the aforementioned technique can be used to spatially pattern different cells types onto the same substrate. Two or more types of chemicals that react specifically to each target cell would be used. As an example, poly(allylamine) containing azidophenyl and *β*-galactose moieties in the side chains (LPAN_3_) was patterned on a photosensitive LPAN_3_-coated PMMA substrate to co-culture hepatocytes and fibroblasts. As a result, hepatocytes and fibroblasts adhered only to the LPAN_3_ and PMMA lane respectively and the co-culture followed the pattern as determined by the photolithrography procedure [223].

### 9.2. Co-Cultivation by Topography Patterning

Compared to chemical patterning, topographic patterning has been less employed. This is partly due to the lack of thorough understanding of cell-topography interaction. Nevertheless, since different cell types have different microtopography preferences, topography patterning can be used to control cell cultures of more than one cell type. For example, when culturing a mixture of cardiac myocytes and fibroblasts on a 10 μm micropegs surface, fibroblasts show a decrease of 50% in proliferation activity. Given that terminally differentiated myocytes do not proliferate but fibroblasts in the primary cultures do, this method effectively keeps fibroblast numbers in control without totally removing them from culture, which is important to maintain long-term survival of myocytes [224].

## 10. Precise Control of Mass Transfer

Compared to traditional cell culture systems, miniaturization of culture devices using MEMS has significantly improved the surface-to-volume ratio. Therefore MEMS devices have better mass transfer capabilities. Moreover, the ability of MEMS to fabricate materials with nanometer resolution means that mass transfer of nutrients and oxygen can be delivered in a precise manner to the cells in culture.

### 10.1. Improved Porosity and Decreased Substrate Thickness

The improvement of matrix porosity and decrease in substrate thickness is important for cells cultured in a sandwich configuration such as hepatocytes. In order to maintain the long-term function of primary hepatocytes, a second layer of collagen has to be overlaid on top of hepatocytes [225]. However, it has been shown that collagen gel itself is a transport barrier for nutrients and metabolic waste [226]. The 100–200 μm thick collagen gel [227] is significantly thicker than the mass transport distance *in vivo* where hepatocytes are generally separated from sinusoids by only 5 μm [228]. A MEMS fabricated silicon nitrate membrane is able to cut down that distance to 3 μm. Moreover, the size and coverage of the pores on the membrane can be precisely determined to achieve optimum mass transfer to cell culture surface ratio [91].

### 10.2. Artificial Vascularization

When culturing a large number of cells for tissue formation, mass transfer poses a great challenge. Few cells can tolerate distances of more than 200 μm from blood or media supply since oxygen consumption rate is much higher than oxygen diffusion rate. Some sensitive cells, such as islets, experience cell death when the diffusion distance is greater than 100 μm [229]. To circumvent this problem, MEMS can be used to create patterns mimicking the branched architecture of vascular and capillary networks.

In [230], trenches were etched on silicon and Pyrex surfaces by using standard photolithography techniques for seeding cells. Endothelial cells were seeded on the trenches while hepatocytes were seeded on the surface. When the co-culture cell sheet was lifted up, a vascularized hepatocyte cell sheet was formed.

In another model, a cell culture chip was separated into media channel and cell culture chamber. The media channel is progressively branched into smaller channels that run beneath the cell culture chamber. Cells seeded in the cell culture chamber were separated from the media channel by a thin layer of membrane. Since the media channel was directly under the cells, effective mass transport of metabolites and small proteins was achieved [231].

## 11. Conclusions

It is clear from these discussions that recreating cell-friendly environments is no small feat. MEMS technology provides an excellent miniaturized platform on which to design and execute specialized microenvironments for biological assays and Tissue Engineering. There are however many parameters that need to be considered in order to create a system optimal to the cell type of interest and the biological question being addressed (basic research), or the engineering assay being developed (applied research). Biocompatibility considerations traditionally address potential toxicity effects from the material surrounding cells, but this consideration is insufficient when designing cell-handling systems. We also highlight here that the ability to control protein adsorption, cell-specific adhesion, orientation, spreading, and long term maintenance also need to be evaluated, and present some methods that serve this purpose. The fairly large repertoire of cell manipulation devices that have been successfully designed and implemented to date, as outlined above, is testament to the utility of MEMS for cellular and Tissue Engineering assays. In moving forward, MEMS technology has the definite potential of allowing researchers to create increasingly complex and detailed modules that would help elucidate the working mechanisms of biological systems.
